# Airway Epithelial Dynamics in Allergy and Related Chronic Inflammatory Airway Diseases

**DOI:** 10.3389/fcell.2020.00204

**Published:** 2020-03-27

**Authors:** Anu Laulajainen-Hongisto, Sanna Katriina Toppila-Salmi, Annika Luukkainen, Robert Kern

**Affiliations:** ^1^Department of Otorhinolaryngology, Helsinki University Hospital, University of Helsinki, Helsinki, Finland; ^2^Laboratory of Cellular and Molecular Immunology, Institute of Microbiology of the Czech Academy of Sciences, Prague, Czechia; ^3^Haartman Institute, Medicum, University of Helsinki, Helsinki, Finland; ^4^Skin and Allergy Hospital, Helsinki University Hospital and University of Helsinki, Helsinki, Finland; ^5^Department of Otolaryngology, Northwestern University Feinberg School of Medicine, Chicago, IL, United States

**Keywords:** asthma, chronic rhinosinusitis, epithelium, allergic rhinitis (AR), inflammation

## Abstract

Allergic rhinitis, chronic rhinosinusitis, and asthma are highly prevalent, multifactorial chronic airway diseases. Several environmental and genetic factors affect airway epithelial dynamics leading to activation of inflammatory mechanisms in the airways. This review links environmental factors to host epithelial immunity in airway diseases. Understanding altered homeostasis of the airway epithelium might provide important targets for diagnostics and therapy of chronic airway diseases.

## Bullet points

Chronic airway diseases are mediated by several, in part unknown, host epithelial- and environment dependent mechanisms.

- The typical disease-leading host-environmental effects are on mucociliary clearance and other innate immunity functions of the epithelial barrier (such as epithelial junctions, pattern recognition, self-renewal, activation of adaptive immunity, metabolism).
- In AR, allergens are able to penetrate airway mucosa by their proteolytic, lipid-binding, and microbial-mimicking properties through the epithelial cells and/or between them.
- Airway viral infections have been implicated in development and exacerbations of AR and asthma, however, their role in CRS pathogenesis remains unclear.
- Airborne irritants, such as cigarette smoke, disrupt epithelial junction proteins and transepithelial resistance, and contribute to CRS alone or together with viral infection.
- Air pollutants might predispose to CRS via aberrant epithelial function.
- Microbiome dysbiosis likely contributes to CRS pathogenesis.
- *S. aureus* colonization indirectly/directly affects mucosal barrier function, leading to a Th2 type inflammation pattern.
- The role of fungi has been shown in at least two CRS phenotypes, fungal balls, and allergic fungal rhinosinusitis (AFRS).

## Introduction

In allergic rhinitis (AR), allergens bind to specific immunoglobulin (Ig)E, leading to rhinorrhea, obstruction, itch, sneeze, and fatigue in sensitized subjects (Wise et al., 2018). AR is interlinked to co-morbidities including asthma, allergic conjunctivitis and atopic dermatitis. However, its role in chronic rhinosinusitis (CRS) is not clear. Chronic inflammation, mucus hypersecretion, edema, variable obstruction, and fatigue characterize asthma. In both children and adults, asthma encompasses different, overlapping phenotypes (Wenzel, 2012; Kaur and Chupp, 2019). Allergic multi-morbidity and predominance in males characterize childhood-onset asthma, whereas adult-onset asthma is more common in females and includes a wide variety of allergic [T helper (Th) Type 2 (Th2)-high] and non-allergic (often Th1-high) phenotypes (Wenzel, 2012; Frohlich et al., 2017). Severe eosinophilic forms, e.g., non-steroidal anti-inflammatory drug (NSAID-) exacerbated respiratory disease (NERD), are more common in adults. So far, only few encouraging signals have been found in asthma prevention. The problem may be over-simplification of terminology. Asthma is not a single disease entity, but rather a complex, heterogeneous, and dynamic immunological disorder strongly influenced by gene – environment interactions.

AR and asthma affect over 300 million people worldwide, thus being major public health problems (Gupta et al., 2004; Nunes et al., 2017; GINA, 2018). The prevalence of AR is 15–50% (Pallasaho et al., 2006; Wiksten et al., 2018), its prevalence at teen-age is 13–38% (Pols et al., 2016; Blaiss et al., 2018; Sterner et al., 2019). The prevalence and socioeconomic impact are difficult to calculate since mild symptoms do not require medical treatment, and most patients outgrow their (especially food) allergies. The prevalence and incidence of, particularly childhood, asthma varies greatly in different parts of the world. After many decades of continuously increasing asthma rates in the Western world, we seem to have reached a plateau in asthma incidence since the beginning of 2000 in many developed countries. In some places even a decrease has been observed. Children migrating from low-income areas to higher socioeconomic areas have a lower prevalence of asthma, suggesting a critical time window for asthma onset in childhood. This suggests the possibility of asthma prevention, since there appear to be predisposing biological factors influenced by the environment. On the other hand, it is likely that within a population, there are genetic factors limiting the number of asthmatics. It should be kept in mind that up to 85% of asthma patients have AR, and on the other hand, 15–38% of AR patients have asthma (Mésidor et al., 2019). Of adults with asthma, 80% have rhinitis, and 50% have chronic rhinosinusitis (Jarvis et al., 2012).

Chronic rhinosinusitis (CRS) is a chronic symptomatic inflammation of the sinonasal tract, with a prevalence of 3–10% (Fokkens et al., 2012; Dietz de Loos et al., 2019; Hirsch et al., 2019). CRS presents with (CRSwNP) or without (CRSsNP) nasal polyps (NP), and is defined by typical subjective symptoms (facial pain, post-nasal drip, obstruction, discharge) lasting for at least 12 weeks, objectively confirmed by either positive endoscopic findings (oedema, mucus secretions, polyps) or positive radiologic findings (mucosal inflammation on sinus CT scans). NERD tends to lead to more severe forms of CRS, with NPs and asthma.

The pathomechanisms of asthma, CRS and AR are related to genetic predisposition and aberrant host-immune interactions during development. The environment strongly affects gene expression by epigenetic mechanisms. In addition to genetic predisposition, climate change, population growth, aging, and urbanization impact the increasing prevalence of chronic airway diseases (Kaur and Chupp, 2019).

Genetics and environmental factors can, during development, significantly modulate barrier homeostasis, influencing the predilection toward chronic airway inflammation. The respiratory epithelium is a part of the innate and adaptive immune system, with responsibility for several functions such as mucociliary clearance, pattern recognition, phagocytosis, antigen presentation, signaling, and self-renewal. Airway epithelial dysfunction is related to several airway diseases. The main focus of this review are the pathomechanisms of human airway epithelium in AR, CRS, and asthma. We also briefly discuss altered airway epithelium in bronchiectasis, primary ciliary dyskinesia (PCD), and cystic fibrosis (CF).

## Genome-Scale Epithelial Factors Behind Airway Diseases

Adult-onset asthma is mediated by activation of molecular pathways leading to persistent mucosal inflammation, variable airway obstruction, inflammation, and tissue remodeling. Genetic and epigenetic variation of the host play key roles (Willis-Owen et al., 2018), and airway dysbiosis may be an important trigger (Huang et al., 2015). Childhood-onset asthma appears to be triggered by allergic and infective immune responses, and barrier dysfunction, with a stronger genetic component and higher heritability (Pividori et al., 2019; Schoettler et al., 2019). Genome-wide association studies (GWAS)s have focused on childhood-onset allergic asthma, and the currently identified single nucleotide polymorphisms (SNP)s thus seem to have lower significance in adult-onset asthma (Pividori et al., 2019). Candidate genes for asthma include interleukin (IL)-4, IL-13 and IL-4R, ADRB2, MS4A2, tumor necrosis factor α (TNFα), cluster of differentiation (CD)14, human leukocyte antigen (HLA)-DRB1 and HLA-DQB1 (Toskala and Kennedy, 2015; Willis-Owen et al., 2018). Combined GWAS and transcriptome-wide association tests have identified several genetic loci that may be relevant in asthma, such as the chromosome 17q locus, HLA, IL-6, interferon regulatory factor 4 (IRF4), chemokine (C-C motif) ligand 20 (CCL20), mucin 5AC (MUC5AC), fatty acid desaturase 2 (FADS2), T-box transcription factor 21 (TBX21), runt-related transcription factor 1 (RUNX1), and cytokine and chemokine receptors and signaling molecules (Pividori et al., 2019; Vercelli and Bleecker, 2019). A study showed that asthma remission is associated with genes related to Th2-mediated inflammation, such as IL1RL1-, IL18R1-, and IL-13 (Vonk et al., 2018). A singular GWAS study of a Japanese adult-onset asthma population showed that SNPs of HLA-, thymic stromal lymphopoietin-WD repeat domain 36 (TSLP-WDR36)-, and ubiquitin specific peptidase 38-GRB2 associated binding protein 1 (USP38-GAB1) loci are associated with adult-onset asthma (Hirota et al., 2011).

A missense variant in arachidonate 15-lipoxygenase (ALOX15) is protective against the development of CRSwNP (Kristjansson et al., 2019). In nasal epithelial cells, IL-13 upregulates ALOX15 and promotes eoxtaxin 3 expression, likely promoting tissue eosinophilia in CRSwNP patients (Li et al., 2019). This pathway may also have a role in epithelial remodeling and barrier dysfunction (Kristjansson et al., 2019). Our transcriptomics of healthy middle turbinate epithelium showed that 75% of protein encoding genes are expressed, suggesting that the epithelium is a very active organ (Hanif et al., 2019). The importance of barrier-environmental interactions was detected also in a single cell transcriptomics study of nasal epithelium, demonstrating that basal cell memory of Type 2 inflammation leads to persistent dysfunction in CRSwNP patients (Ordovas-Montanes et al., 2018).

Multi-gene expression-based biomarkers of asthma were studied in a Network-identified Transcription Factor –framework (Ahsen et al., 2019). ETS translocation variant (ETV4) and Peroxisome proliferator-activated receptor gamma (PPARG) were identified as being the most significant transcription factors. The group further performed validation studies using a nasal epithelium cell line in which both transcription factors were knocked down by siRNA. The respective cell lines produced a significantly decreased amount of IL-8 and IL-6, before poly (I:C) stimulation and before (Ahsen et al., 2019).

## Epithelial Dynamics in Upper Airway Diseases

**FIGURE 1:**
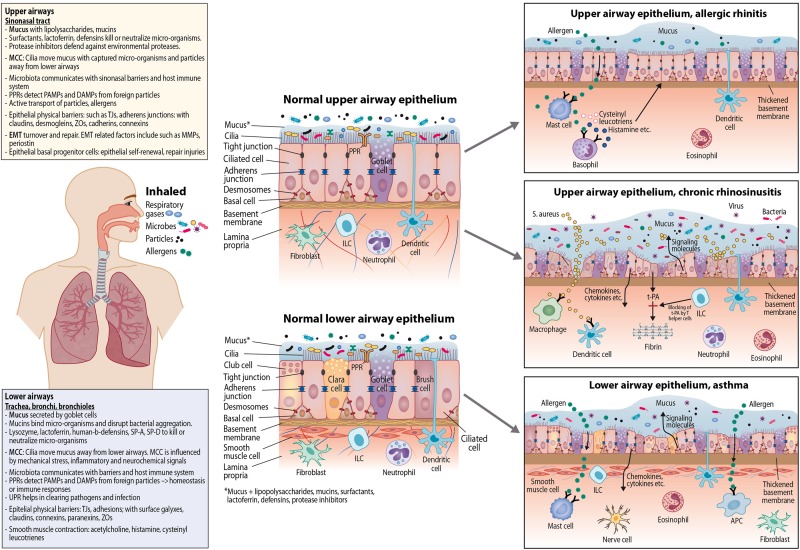
Epithelial dynamics in upper and lower airway epithelium; normal epithelium and epithelium in allergic rhinitis, chronic rhinosinusitis, and asthma. APC, Antigen-presenting cell; ILC, innate lymphoid cell; PPR, pattern recognition receptor; PAMP, pathogen-associated molecular pattern; DAMP, danger-associated molecular pattern; MCC, mucociliary clearance; TJ, tight junction; t-PA, tissue plasminogen activator; ZO, zonula occludin; EMT, epithelial-mesenchymal transition; MMP, matrix metalloproteinase; SP, surfactant protein; UPR, unfolded protein response.

Airway barriers likely have a key role in the development of CRS and AR. In AR, mucosal inflammation is triggered by allergens, together with other environmental factors, leading to IgE-mediated mucosal inflammation. In CRS, mucosal inflammation is triggered by a dysfunctional interaction between exogenous agents and the immune system (Liao et al., 2014; Lam et al., 2015; Hoggard et al., 2017). Compared to AR, there is larger inter- and intra-individual variation in the causal factors behind CRS (Figure 1).

### Environmental Factors and Upper Airway Epithelium

Allergen entry into airway mucosa is facilitated by several allergen-, host-, and environment-dependent mechanisms. Allergens are able to penetrate airway mucosa by their proteolytic, lipid-binding, and microbial-mimicking properties (Toppila-Salmi et al., 2015). Mite allergen entry has shown to be facilitated by altered pattern recognition pathways (Lam et al., 2015). Our study group has shown that birch pollen allergens (Bet v1) are able to bind plasma membrane lipid rafts (Toppila-Salmi et al., 2015) and to be transported in caveolar vesicles through the nasal epithelium until they reach mast cells. However, this was detected in allergic patients only (Joenvaara et al., 2009). Moreover, natural birch exposure causes transcriptomics alterations in controls; the fold changes were detected in the nasal epithelial transcripts belonging to Gene Ontology (GO) –category “Immunology”. In contrast, transcripts of the AR group were enriched in “Response to virus” and “Cellular transportation” –categories (Mattila et al., 2010). Similar categories have been demonstrated in grass pollen allergy (Roschmann et al., 2011). We performed whole transcriptomic sequencing of nasal epithelial brush samples and demonstrated that birch pollen allergen immunotherapy alters the transcript profile during pollen exposure season toward that of control samples (Hanif et al., 2019). Moreover, the data showed that in AR patients who started with subcutaneous birch pollen immunotherapy, microbiome diversity of nasal epithelium shifted toward that of controls (Hanif et al., 2019).

AR has been shown to associated with active/passive smoking (Saulyte et al., 2014). Taken together, in AR, airborne allergens, as well as tobacco smoke, microbes, and air pollutants are related to the development and aggravation of the disease (Saulyte et al., 2014; Wiksten et al., 2018).

In prevention of CRS development and its progression, commensal organisms likely play a protective role by preventing mucosal colonization by pathogens and putatively providing metabolites improving mucosal health. It has been assumed that microbial agents and microbiome dysbiosis are some of the most important drivers of CRS pathogenesis. *S. aureus* can directly affect mucosal barrier function and drive Type 2 inflammation (Ryu and Kim, 2020). Molecular sequencing techniques are evolving in power and enable studies on total and relative microbial abundance and their functional activity within the sinonasal tract (Earl et al., 2018). Yet it is important to note intra- and inter-individual variation of the samples and sites of sampling (Copeland et al., 2018). In a cross-sectional study involving sequencing of the V3-V4 region of the 16S ribosomal RNA (rRNA) gene in nasal samples, decreased middle meatal microbiome diversity was detected in AR, CRSsNP, and CRSwNP groups compared to controls, with high variability in microbe profiles and even within subjects (Lal et al., 2017). Hence, recent studies support the hypothesis that dysbiosis of the microbiome may trigger mucosal inflammation both in AR and in CRS (Knight et al., 2018). Airway viral infections have been implicated in development and exacerbations of AR and asthma, however, their role in CRS pathogenesis remains unclear. In addition, the role of fungi has been shown in at least two CRS phenotypes, fungal balls and allergic fungal rhinosinusitis (AFRS).

Cigarette smoke extract has been shown to disrupt epithelial junction proteins and transepithelial resistance in an epithelial sinonasal model derived from control patient samples (Tharakan et al., 2016). In addition, cigarette smoke and viral infection might contribute to polyp remodeling (Yamin et al., 2015). Decreased levels of airway epithelial clara cell protein 16 (CC16) are found in the nasal secretions and plasma of patients with CRS and in subjects exposed to high levels of air pollutants (Peric et al., 2018). There is some evidence that heavy metal exposure is related to CRS (Khlifi et al., 2015).

### Upper Airway Epithelial Functions During AR and CRS

The upper airway barrier provides several important innate immunity functions, blockage of microbe entry, and recruitment of leukocytes. Airway epithelium plays a critical role in conducting and humidifying air, responding to trigeminal and olfactory stimuli, and in host defense. The airway epithelial barrier comprises ciliated cells, olfactory cells (in olfactory epithelium), mucus-secreting goblet cells, basal cells with progenitor capacity (Bravo et al., 2013) and few chemosensory cells (Kohanski et al., 2018).

Motile cilia consist of microtubules and dynein arms, powered by adenosine triphosphate (ATP) (Knowles et al., 2013; Popatia et al., 2014). Coordinated ciliary beating transports debris-laden mucus from respiratory passages toward the oropharynx (Toppila-Salmi et al., 2015). Genetic and acquired defects in mucociliary flow with increased mucus viscosity are associated with a high incidence of CRS (Cutting, 2005; Gudis et al., 2012). Cilia are coated with mucins and tethered mucopolysaccharides (Toppila-Salmi et al., 2015). Li Y. Y. et al. (2014) detected cilia abnormalities in CRSwNP (Jiao et al., 2015), and that impaired ciliated epithelial differentiation may be mediated via decreased interferon gamma (IFNγ) and IL-13 levels leading to secondary declines in ciliary beat frequency (Jiao et al., 2015). Reduced expression of WDPCP, a ciliogenesis protein, due to inflammatory cytokines resulted in impaired ciliogenesis and cilia function in CRS patients (Ma et al., 2017). Secretory cells produce polymeric gel-forming mucins, such as MUC5AC and MUC5B (Toppila-Salmi et al., 2015). Gel-forming mucins are secreted into the airway lumen and are responsible for the characteristic viscoelastic properties of the mucus gel layer. Tipirneni et al. (2018) identified that dynamic mucus strand velocities from submucosal glands, a major component of mucociliary clearance, were significantly decreased in CRS. Surfactant protein A gene expression has been shown to be increased in CRS and decreased in primary atrophic rhinitis (El-Anwar et al., 2015). In overview, CRS is commonly associated with mucociliary dysfunction.

Both epithelial specification and terminal differentiation are critical to epithelial homeostasis in airway diseases (Ordovas-Montanes et al., 2018). Environmental and mucosal signals regulate epithelial stem-cell self-renewal under normal conditions (Yilmaz et al., 2012) and in CRS (Ordovas-Montanes et al., 2018). Under physiological conditions, environmental and intrinsic signals are able to rapidly alter the composition and function of the epithelium. A study group performed single cell transcriptomics of polyps/scraping epithelium of twelve CRSwNP and nine control subjects. They detected differences in expression of antimicrobial genes by secretory cells, a loss of glandular cell heterogeneity, and that polyp basal progenitor cells were locked to a Type 2 memory (Ordovas-Montanes et al., 2018). Epithelial basal progenitor cells are able to migrate and proliferate into ciliated and goblet cells in injured regions.

Upper airway epithelium secretes several cytokines including TSLP, IL-33, and IL-25. These cytokines are released by tissue damage, pathogen recognition or allergen exposure. They effect Th2 cell function either directly or via innate lymphoid cells (ILCs), which in turn produce IL-5, IL-9, and IL-13 (Licona-Limon et al., 2013; Scadding, 2014), related to Th2-type CRS, asthma and AR (Toppila-Salmi et al., 2015). *S. aureus* colonization is more common in patients with CRSwNP than controls (Tomassen et al., 2016). *S. aureus* produces serine protease-like protein (Spl), which causes Th2- biased inflammatory responses via TSLP and IL-33 (Stentzel et al., 2017; Ryu and Kim, 2020). *S. aureus* specific IgE has been associated with both CRSwNP and asthma. *S. aureus* enterotoxins and *S. aureus* Spls have been found to have allergenic properties (Stentzel et al., 2017; Ryu and Kim, 2020). Type 2 cytokines inhibit t-PA (tissue plasminogen activator) resulting in the deposition of fibrin mesh to form the tissue matrix of NPs (Takabayashi et al., 2013).

Pattern recognition receptors (PRRs) rapidly detect microbial and other foreign molecular patterns and either maintain homoeostasis or induce immune responses. Luukkainen et al. (2018) stimulated a co-culture of peripheral blood mononuclear cells and nasal epithelium, differentiated from stem/progenitor cells, with H3N2-virus and detected rapid activation of monocytes, natural killer (NK)-cells and innate T-cells (MAIT and γδ T cells). We demonstrated high baseline nasal epithelial expression of Toll Like receptor (TLR) proteins (TLR1-7, TLR9-10) and MyD88 both in AR and in controls (Renkonen et al., 2015). After off-seasonal intranasal birch pollen challenge, a negative change in the expression score of TLR1 and TLR6 proteins was detected in the atopic group. Tengroth et al. (2014b) demonstrated abundant TLR3, TLR7, TLR9, RIG-I, and MDA-5 in nasal epithelium. The group detected defects in TLR9-mediated microbial defense in CRSwNP (Tengroth et al., 2014a). Studies on polyp and control tissue show that increased epithelial TLR2 (Sun et al., 2012) and TLR4 may be related with CRSwNP (Sun et al., 2012; Shimizu et al., 2016; Hu and Li, 2018). Nasal polyp fibroblast activation may occur via TLR2 (Shin et al., 2016a, b; Tsai et al., 2018), TLR4 (Cho et al., 2014, 2016), TLR5 (Shin et al., 2016b), and TLR9 (Park et al., 2018) might be related to polyp B-cell activation (Xu et al., 2014). NOD family PRRs form a major component of the inflammasome, and are related in programmed pro-inflammatory cell death distinct from apoptosis. Jardeleza et al. (2013) detected involvement of inflammasome complexes and their signaling pathways in *Staphylococcus aureus* -biofilm positive CRSwNP. Lin et al. (2016) detected increased NLRP3 and caspase-1 in eosinophilic CRSwNP, and augmented inflammasome signaling pathway by lipopolysaccharides (LPS). Bitter taste receptors (T2Rs) are G protein-coupled receptors that function as non-classical PRRs. Bacterial quinolones and acyl-homoserine lactones, secreted by gram-negative bacteria, can activate airway T2R-mediated immune responses (Lee and Cohen, 2014; Freund et al., 2018). Linkage studies have demonstrated associations between taste receptor genetics with CRS (Cohen, 2017).

Airway epithelium secretes defense molecules such as surfactant, lactoferrin, and defensins, which kill or neutralize microorganisms. Some evidence suggests that decreased secretion of host defense molecules is associated with CRS (Tieu et al., 2010). Decreased expression levels of palate, lung, and nasal epithelium clone protein (PLUNC), and increased surfactant-B and alpha-defensin levels have been observed in CRSwNP, possibly secondary to loss of glands (Seshadri et al., 2012; Jardeleza et al., 2013; Lin et al., 2016; Tsybikov et al., 2016).

Protease inhibitors regulate environmental proteases that might compromise barrier integrity. Blocking protease allergens with inhibitors reduces allergic responses in AR (Suzuki et al., 2006). Fukuoka et al. (2019) showed that human cystatin SN, an endogenous protease inhibitor, suppresses AR symptoms by inhibiting allergen protease activities and by allergen-specifically protecting nasal TJ barrier. Kouzaki et al. (2016) showed lower host expression of two protease inhibitors (cystatin A and SPINK5) in nasal epithelial cells extracted from patients with eosinophilic CRS compared with control and non-eosinophilic CRS groups. This suggests that an imbalance of proteases and protease inhibitors within the epithelial barrier may contribute to the pathogenesis of Type 2 diseases in general (Wu et al., 2018).

Epithelial cells undergo turnover and repair after injury through epithelial to mesenchymal transition (EMT), with a rapid and normally reversible modulation of the epithelial phenotype toward mesenchymal cells (Toppila-Salmi et al., 2015). During EMT, epithelial cells lose cell-cell polarity and adhesion to become migratory. They get mesenchymal features such as alpha-smooth muscle actin, vimentin, matrix metalloproteinases (MMPs), and transcription factors. In CRS, aberrant epithelial structure and function may lead to increased permeability to foreign material suggesting this as an early factor in CRS pathogenesis (Pothoven et al., 2015; Ramezanpour et al., 2016; Suzuki et al., 2016; Jiao et al., 2019). Inflammation leads to remodeling with cytokines, mediators, enzymes, and other factors determining the remodeling pattern, not fully depending on the CRS phenotype. The duration and type of inflammation affect mucosal structure and function, and clinical severity of inflammation. Remodeling changes of CRS include fibrosis, basement membrane thickening (BMT), goblet cell hyperplasia, epithelial barrier abnormalities and polyp formation, osteitis, and angiogenesis (Barham et al., 2015; Kuhar et al., 2017).

Periostin promotes adhesion and migration of epithelial cells and is associated with CRSwNP (Ishida et al., 2012; Ohta et al., 2014; Laury et al., 2015; Milonski et al., 2015; Shiono et al., 2015; Wang et al., 2015; Ebenezer et al., 2017; Xu et al., 2017; Wei et al., 2018; Yang et al., 2018; Lehmann et al., 2019) and asthma (Carpagnano et al., 2018; Wei et al., 2018). The EMT process is driven by an array of factors such as WNT, reactive oxygen species, proteases, HIF1, IL-13 Epiregulin, Oncostatin M, and IL-1 (Oyer et al., 2013; Batzakakis et al., 2014; Schleimer, 2017; Aazami et al., 2018; Yang et al., 2018; Tomaszewska et al., 2019). CRSwNP patients have altered expression levels of EMT related factors, such as MMP-1 (Malinsky et al., 2013; Homma et al., 2017), MMP-2 (Malinsky et al., 2013), TIMP-1 (Wang et al., 2012; Muluk et al., 2015), MMP-7 (Yang et al., 2017; Chen et al., 2018), MMP-9 (Wang et al., 2012; Malinsky et al., 2013; Yeo et al., 2013; Li et al., 2015; Muluk et al., 2015; Chen et al., 2018; Suzuki et al., 2018; Xiang et al., 2019), TIMP-2 (Li et al., 2015) E-cadherin (Hupin et al., 2014; Kim et al., 2018; Deng et al., 2019). BMT is associated with duration of inflammation independent of tissue eosinophilia (Kountakis et al., 2004; Kuhar et al., 2017). TGF-β has been most closely linked to fibrosis, but IL-13 and osteopontin have also been implicated in BMT and fibrosis (Rehl et al., 2007; Van Bruaene and Bachert, 2011; Shi et al., 2013; Schleimer and Berdnikovs, 2017). Abnormalities of the coagulation cascade have also been associated with polyp formation including Factor X, tissue factor and thrombin (Shimizu et al., 2015; Shimizu et al., 2017; Takabayashi et al., 2019). There is some evidence that stem cells in the epithelium maintain a memory for the chronic immature EMT state in severe Type 2 CRS (Lehmann et al., 2019), promoting barrier failure, antigen access, and this inflammation (Pothoven and Schleimer, 2017). Type 2 cytokines inhibit t-PA activity so in the presence of high levels of Type 2 inflammation, the matrix will be retained and grow (Peterson et al., 2012).

Compared to the CRS phenotype, remodeling has been less studied in patients with AR. Li and Li (2019) performed transmission electron microscopy, western blot, and qPCR to nasal epithelial samples of patients with AR and detected increased autophagosomes, Beclin-1, LC3-II, and Collagen III, along with increased symptom scores, suggesting a link between autophagy and airway remodeling in AR. Taken together, several epithelial phenomena take part in the development and chronicity of the inflammation during CRS and AR.

Epithelial physical barriers are maintained by intercellular junctions. Tight junctions (TJ) are located the most apically, are linked to the cytoskeleton and inhibit solute and water movement through the paracellular space, thus establishing cell polarity (Toppila-Salmi et al., 2015). Inhaled allergens, microbial or viral infections, cytokines, hypoxia, and zinc deficiency are able to affect TJ molecules and epithelial barrier function in the airways (Roscioli et al., 2017; Jiao et al., 2019). Several genes/molecules, such as SPINK5, S100A7, S100A8/9, PCDH1, NDRG1, SPRR, and p63 are involved in modulating the physical barrier function in CRS (Jiao et al., 2019). Yu et al. (2013) demonstrated decreased expression of Epithelium membrane protein 1 (EMP1), a TJ protein, in nasal polyp epithelium compared to control nasal mucosa. Soyka et al. (2012) detected a decreased trans-tissue resistance in biopsy specimens from patients with CRSwNP and also decreased TJ proteins. Suzuki et al. (2016) detected that nasal polyp had a higher expression of claudin (Cld)1 but lower expression of tricellulin compared with the turbinate. Integrity of the nasal epithelial TJ barrier has been shown to be compromised in Chinese patients with eosinophilic and non-eosinophilic CRSwNP transforming growth factor (TGF)-β1 seems to plays an important role in inducing TJ barrier defects (Jiao et al., 2018). Li et al. showed decreased expression of epithelial zonula occludin (ZO)-1, Cld1, desmoglein (DSG)1, and DSG2 in CRSwNP and decreased expression of Cld1, DSG1, and DSG2 in CRSsNP (Li Y. et al., 2014).

Adherens junctions are located more basally than TJs. Epithelial cadherin (E-cadherin) creates intercellular interactions. Together, these junction proteins function to limit intercellular passage of fluid and protect the underlying tissue from exposure to noxious and allergenic stimuli (London and Ramanathan, 2017). Epithelial cell communication is mediated via Gap junction channels, which are formed by connexin proteins enabling cell communication (Kim et al., 2016). Expression of connexins have been demonstrated to be increased in CRS compared to controls (Kim et al., 2016). Hence, epithelial barrier dysfunction may contribute to AR and CRS through allowing increased passage of antigens and exposure of underlying tissue to these stimuli (Toppila-Salmi et al., 2015; London and Ramanathan, 2017).

## Epithelial Dynamics in Lower Airway Diseases

Ventilation moves air through the conducting airways to and from the alveoli. The inhaled air contains respiratory gases, particles, microbes, and toxins. Defense mechanisms preventing the entry of unwanted substances into the lung tissue and circulatory system include the branching structure of the conducting airways, the layers of mucus covering the airways, mucociliary clearance, contraction of the airway smooth muscle, the tight adhesions of airway epithelial cells (AEC) and their underlying stroma, and the production of host-defense molecules regulated by exposure to toxins, pathogens and cytokines (Lambrecht and Hammad, 2012; Whitsett and Alenghat, 2015).

Asthma is a chronic pulmonary disease characterized by airway inflammation, airway hyperreactivity, and recurrent, reversible airway obstruction. Several asthma phenotypes related to disease mechanisms exist; these include childhood-onset allergic asthma, adult-onset eosinophilic asthma, obesity-related asthma, and neutrophilic asthma (Wenzel, 2012). Airway inflammation is initiated by AECs as a defense against inhaled pathogens and particles, e.g., allergens. In asthma these defense mechanisms are hyperreactive. Excessive mucus production contributes to airway obstruction, which is mainly caused by contraction of airway smooth muscle (Erle and Sheppard, 2014; Figure 1).

### Environmental Factors, Epithelium, and Asthma

The risk factors for asthma include AR and allergic conjunctivitis, atopic dermatitis, exposure to air pollution, cigarette smoke, occupational risk factors, obesity, and genetic factors (Polosa and Thomson, 2013; Ilmarinen et al., 2015; Toskala and Kennedy, 2015; Willis-Owen et al., 2018; Toppila-Salmi et al., 2019). Childhood-onset asthma has a stronger genetic component than adult asthma and is triggered due to dysregulated allergy and epithelial barrier function (Pividori et al., 2019; Schoettler et al., 2019). The development of adult asthma is more likely in patients with an accumulation of several risk factors and with allergic multimorbidities (Hallit et al., 2019; Pividori et al., 2019; Toppila-Salmi et al., 2019). Asthma, rhinitis, and chronic rhinosinusitis often co-exist (Jarvis et al., 2012).

The airway microbiome communicates with the respiratory epithelium and has an important role in maintaining airway health (Hansel et al., 2013; Morris et al., 2013; Huang et al., 2015). Disruption of normal mucociliary clearance in smokers, and in patients with asthma or cystic fibrosis (CF) affects airway microbiome homeostasis and may lead to disease (Dickson et al., 2013; Hansel et al., 2013; Morris et al., 2013). Early childhood respiratory tract microbial exposure influences immune responses, regulating Th1 and Th2 immunity and affecting future asthmatic responses (Busse et al., 2010; Ege et al., 2011). In children with a family history of asthma, the risk of asthma is increased by severe respiratory syncytial virus (RSV) infections (Sigurs et al., 2000). Virus infections of the respiratory tract, especially caused by rhinoviruses, associate with asthma exacerbations (Busse et al., 2010). Patients with asthma may have deficient IFNγ response leading to prolonged and more severe viral infections, deficient IFNγ response has also been linked to Th2 type immune reaction (Lisspers et al., 2018).

### Epithelial Functions in Asthma

The trachea, bronchi, and bronchioles are mainly lined by ciliated pseudostratified epithelium. Also serous, club, goblet and neuroendocrine cells, and smooth muscle cells are found in the airways.

In patients with asthma and bronchiectasis, excessive goblet cell differentiation, and mucus production are common (Whitsett and Alenghat, 2015).

Submucosal glands in the trachea and bronchi are lined by basal, ciliated, myoepithelial, serous and goblet cells, and secrete fluids and host-defense molecules such as lysozyme, lactoferrin, human-b-defensins, and surfactant proteins A and D. Mucins are large glycoproteins mainly produced by goblet cells (mucin granules), they have important functions in cell-cell interaction, EGFR signaling and airway protection (Thai et al., 2008; Voynow and Rubin, 2009). Mucins tied to AECs (MUC4, MUC13, MUC16, MUC21) create a barrier that by pathogen or host-associated proteases can shed, enabling the unwanted microbe to be removed by mucociliary clearance. Secreted airway mucins (MUC5B, MUC5AC, and MUC2) form a microbe binding, bacterial aggregation disrupting gel. The expression of secreted mucins is induced by transcription factors e.g., MAPK, STAT6, and inhibited by transcription factors FOXa2 and TTF-1 (Maeda et al., 2011; Alevy et al., 2012; Whitsett and Alenghat, 2015).

Dysfunctional movement of the cilia leads to impaired mucociliary clearance, accumulation of thick mucus and recurrent infections. Primary ciliary dyskinesia (PCD) patients have structurally abnormal cilia, decreased production of nitric oxide by AECs, bronchiectasis, and chronic bacterial infections of both the upper and lower airways (Knowles et al., 2013; Popatia et al., 2014). Secondary impairment of ciliary movement, e.g., due to smoking or CF, leads to abnormalities in airway hydration and mucus production. Ciliary function is influenced by mechanical stress, inflammatory and neurochemical signals, including paracrine signals of AECs. The gap junctions between cells exchange responses induced by these stimuli, connexin Cnx43 plays an important role in this (Martin and Prince, 2008; Bou Saab et al., 2014).

The respiratory epithelium recognizes pathogen-associated molecular patterns (PAMP), from commensal microbes or pathogens, and danger-associated molecular patterns (DAMP), from cell stress or cell death. Membrane associated or cytosolic PRRs expressed in AECs recognize PAMPs and DAMPs, resulting in signaling via TLRs, mitogen-activated protein kinase (MAPK), IRF and nuclear factor-κB (NF-κB) family transcription factors, reactive oxygen species (ROS), and Janus kinase – signal transducer and activator of transcription (JAK-STAT) signaling pathway, some of which also interact (Athari, 2019). The resulting cytokine and chemokine signaling and production of antimicrobial proteins leads to recruitment and activation of innate and adaptive immune system cells, and regulation of barrier function (Voynow and Rubin, 2009; Lambrecht and Hammad, 2012; Erle and Sheppard, 2014; Whitsett and Alenghat, 2015). Also unfolded protein response (UPR) helps in clearing pathogens and infection by linking the synthesis of misfolded proteins encoded by pathogens to inflammatory signaling, activating apoptosis and necrosis to remove the pathogens and infected epithelial cells (Osorio et al., 2013). Alveolar macrophages, but also AECs ingest infected and apoptotic cells by phagocytosis (Juncadella et al., 2013).

TLR4 can be activated by LPS, cigarette smoke, RSV, and inflammatory cytokines (Monick et al., 2003; Armstrong et al., 2004; Pace et al., 2008). Allergens cause TLR4-dependent activation of NF-κBs, resulting in secretion of chemokines and cytokines, such as IL-33, TSLP, and IL-25, and activation and recruitment of pulmonary dendritic cells, Type 2 innate lymphoid cells (ILC2 cells) and Th2 lymphocytes (Hammad et al., 2009; Tan et al., 2010; Lambrecht and Hammad, 2014). Interferons are secreted to defend against viral infections, and in patients with asthma, deficient IFNγ response may lead to prolonged and more severe viral infections (Lisspers et al., 2018). A study group performed expression profiling of airway epithelium that demonstrated similar cytokine profiles of EGR1, DUSP1, FOSL1, JUN, MYC, and IL-6 after stimulation of AECs with either dsRNA or with house dust mite, however, both triggers also induced a specific response (e.g., ATF3, FOS, and NF-κB1) (Golebski et al., 2014). It could thus be possible that microbial infection and its underlying immune dysfunction might be a phenotypic or clinical feature of both atopic and non-atopic chronic conditions in the airways rather than only a secondary effect (Juhn, 2014).

Epithelial extracellular vesicles (EVs) can transfer microRNAs (miRNAs). In particular miR-34a, miR-92b, and miR-210 may have a role in the development of the Th 2 response in asthma (Bartel et al., 2019). As a sign of epithelial dysfunction in asthma, AEC DNA methylation is different in asthmatics vs. non-asthmatics (Clifford et al., 2019).

A recent study shows that asthmatic Type 2 inflammation and airway hyperresponsiveness are related to mast cell infiltration into the airway epithelium and that AECs and mast cells communicate through IL-33 signaling that regulates inflammation (Altman et al., 2019). In allergic asthma, the inflammatory process is initiated by Th2 cells that produce cytokines, such as IL-4, IL-5, IL-9, IL-13, leading to the production of IgE, eosinophilia, and goblet cell hyperplasia (Whitsett and Alenghat, 2015; Athari, 2019). Pathways to regulate goblet cell hyperplasia are also activated by epidermal growth factor receptor (EGFR) (Tyner et al., 2006). Contraction of the airway smooth muscle, resulting in airway narrowing, is induced by acetylcholine released from efferent parasympathetic nerves, or by histamine and cysteinyl leukotrienes released from mast cells and basophils (Erle and Sheppard, 2014). Asthma is modulated also by Th9 and Th17 cells, that produce IL-17F, IL-22, and IL-17A inducing airway inflammation and enhancing smooth muscle contractility (Kudo et al., 2013). Airway epithelium and smooth muscle cells communicate also via epithelial cell-derived endothelin-1 (Lan et al., 2018). Airway hyperresponsiveness is linked to IL-4 (Athari, 2019), and also to IL-13 and IL-17 by inducing RhoA expression, and Ca^2+^ sensitization of bronchial smooth muscle (Erle and Sheppard, 2014). Airway smooth muscle contractibility is also increased by TNFα (Goto et al., 2009).

The barriers created by AECs are important for the health and normal function of the airways. Increased epithelial permeability and inflammation with disruptions in the TJ complexes may affect the pathogenesis of asthma and other pulmonary diseases (Georas and Rezaee, 2014). Barriers of AECs include secretory products, surface glycocalyces, membranes and intracellular junction proteins consisting of claudins, connexins, paranexins, adhesions, and ZOs. Pulmonary cells claudins, such as claudin 3 (Cld3), Cld4, and Cld18 create interlocking structures forming these TJs (Whitsett and Alenghat, 2015).

The alveoli are lined by only two cell types, simple squamous alveolar squamous epithelium type I (90%) or cuboidal type II cells. The type II alveolar cells are the main progenitor cells for alveolar cells. The type I alveolar cells are in close interaction with the pulmonary capillary endothelial cells to ensure exchange of respiratory gases. Surfactant lipids and proteins secreted by type II epithelial cells diminish alveolar surface tension. They ensure optimal surface tension, prevention of alveolar collapse but ensuring elasticity and gas exchange. The type II alveolar cells have many lipid-rich lamellar bodies, microvilli on the apical surfaces and express surfactant homeostasis mediating lipids and proteins, such as surfactant protein (SP)-A, SP-B, SP-C, SP-D5. SPs bind, aggregate and/or directly kill microbial pathogens, enhancing their clearance by immune system cells (Numata et al., 2010; Whitsett et al., 2010). SP-A and SP-D belong to collectins (innate host-defense proteins); they bind with PAMPs. SP-A and SP-B form tubular myelin that together with lipids forms a pool of surfactant and host-defense proteins (e.g., lysozymes and SP-C). This enhances opsonization and killing of pathogens by alveolar macrophages and regulates macrophage, neutrophil, and lymphocyte activity (Hartshorn, 2010; Whitsett and Alenghat, 2015). Loss of SP-B or SP-C production impairs both alveolar barrier and macrophage functions and causes tissue injury and inflammation (Akei et al., 2006). The volume and composition of surfactant pools are maintained by *de novo* synthesis, reuptake and recycling by alveolar type II cells, also by the catabolic activity of alveolar macrophages, in processes partly regulated by granulocyte-macrophage colony-stimulating factors (GM-CSF) (Whitsett et al., 2010; Whitsett and Alenghat, 2015). Impairment of GM-CSF signaling has been associated with susceptibility to bacterial and viral infections (Tourdot et al., 2008).

Two types of macrophages can be found in the lungs, alveolar macrophages and interstitial macrophages (Jiang and Zhu, 2016). Macrophages can roughly be classified to classically activated (M1) and alternatively activated (M2) macrophages, mirroring Th1 and Th2 polarization of T cells. M1 macrophages are induced by INFγ and LPS, they are involved in pathogen clearance. M2 macrophages are induced by IL-4 and IL-13, and are involved in wound healing and anti-inflammatory responses (Sica and Mantovani, 2012; Liu et al., 2013; Shapouri-Moghaddam et al., 2018). It is possible that M1-mediated inflammation in the adipose tissue of obese patients enhances M2-mediated asthmatic inflammation of the lungs (Sharma et al., 2017). CD163 is a known marker for M2 macrophages, it seems to have a role in airway hyperresponsiveness and asthma (Jiang and Zhu, 2016; Tokunaga et al., 2019). Alveolar macrophages respond to pathogens, sense antigens and activate innate and acquired immunity. During infection, they clear apoptotic cells and inhibit inflammation (Westphalen et al., 2014). Alveolar epithelial cells and macrophages directly communicate via Cnx43 channels to modify inflammatory signals and regulate cytokine and chemokine expression in response to pathogens (Westphalen et al., 2014; Whitsett and Alenghat, 2015). Interventions to modulate phenotypes of alveolar macrophages may have therapeutic potential in the treatment of asthma (Jiang and Zhu, 2016).

### Epithelial Functions in Bronchiectasis, Primary Ciliary Dyskinesia, and Cystic Fibrosis

Bronchiectasis results from severe airway infection and inflammation. Its pathomechanisms are in part unknown. Patients with bronchiectasis have irreversible bronchial dilatation with excessive goblet cell differentiation and mucus production (Whitsett and Alenghat, 2015). Defective airway host-defense, infections and inflammation contribute to the development of bronchiectasis (Guan et al., 2018).

In PCD patients, the dysfunctional movement of cilia leads to impaired mucociliary clearance, accumulation of thick mucus, recurrent infections, and may lead to the development of bronchiectasis with chronic bacterial infections (Knowles et al., 2013; Popatia et al., 2014). Chronic infections occur both in the lower and upper airways. CRS and CRSwNP are common in patients with bronchiectasis according to a Spanish study (Guilemany et al., 2009). However, Chinese adults with bronchiectasis seem to have less CRS than in western populations (Guan et al., 2015).

Bronchiectasis is also a common manifestation of cystic fibrosis (CF) (Guan et al., 2018). Patients with CF have mutations in the gene encoding cystic fibrosis transmembrane conductance regulator (CFTR), leading to inhibition of Cl^–^ and HCO_3_^–^ transport by airway and submucosal gland epithelial cells. This results in thickening of mucus, which leads to flawed mucociliary clearance, secondary impairment in ciliary movement and chronic bacterial infection (Whitsett and Alenghat, 2015), both in lungs and paranasal sinuses (Hamilos, 2016). CRSwNP occurs with increased prevalence in CF patients (Hamilos, 2016). There is evidence that increased intracellular levels of Cl^–^ may be associated with chronic inflammation in bronchiectasis and CF (Zhang et al., 2018).

There is a strong association of a homozygous mutation of the chloride transport gene (CFTR) with childhood-onset CRS (Kim and Ober, 2019). Heterozygous CFTR mutations are also associated with CRS signals, albeit less than in clinical CF, with disease usually presenting in adulthood (Hsu et al., 2013; Yoo and Suh, 2017). The impaired mucociliary flow seen with CFTR mutations is presumed to intensify microbial exposure, also affecting disease course (Wang et al., 2000). The importance of CFTR in childhood-onset CRS suggests that also other barrier-related genes might play a role in CRS initiation.

Much of the non-protein encoding DNA encodes functional RNAs, important in gene regulation. A microarray analysis of bronchial brushings showed that 1,063 out of over 30,000 long non-coding RNA transcripts had different expression between CF and non-CF individuals, the pathologic processes in CF patients’ airway epithelium are thus possibly partly driven by non-coding RNAs, possibly altering gene expression regulation (McKiernan et al., 2014).

## Epithelium and United Airway Concept

Most genes related to viral responses were similarly induced in upper and lower airways, in a study involving poly (I:C) -stimulated intra-individual primary nasal and bronchial epithelial cells, however, asthma patients had impaired induction of several interferon-related genes (Wagener et al., 2014). In a cross-sectional study of asthmatic children; transcriptomic sequencing was performed in 10 children and targeted sequencing in 40 children. Expression profiles reassembled in nasal and bronchial brushings, were specific to asthma independently of atopic status, and clustering analysis identified Th2-high and low subjects differentiated by expression of 70 genes (such as IL-13, IL-5, periostin, CLCA1, SERPINB2) (Poole et al., 2014). These Th2-high subjects more likely had atopy, atopic asthma, eosinophilia, and rhinitis. Hence, using less invasive nasal brushing samples and epithelial profiling may be clinically applicable when assessing asthma endotype and specific treatment. There is evidence that nasal epithelial cells could act as surrogates to bronchial epithelial cells in studies investigating airway inflammation (McDougall et al., 2008; Roberts et al., 2018). This is, however, not always feasible, bronchial epithelial cells are more susceptible to rhinovirus infection than nasal epithelial cells (Lopez-Souza et al., 2009).

## Conclusion and Future Needs

Airway epithelium has important innate immune functions, all these functions seem to be essentially involved in the development of AR, CRS, and asthma.

Under normal conditions, several airborne factors are inhaled through the respiratory system and they interact with the airway barriers. In healthy individuals, the airway barrier limits the entry of pathogens and allergens, regulates the interaction with the host immune system promoting homeostasis. In AR, CRS, and asthma, barrier penetration results in inflammatory responses that are dynamic and heterogeneous and not clearly matched to the inciting agents. Asthma is not a single disease entity, and childhood-onset and adult-onset asthma have different backgrounds regarding their genetics, association with AR and CRS, possibly also differences in microbe-host interactions. Currently, only triggering factors of AR and other allergic diseases are known. Of note, allergens have an impact in pathogenesis and exacerbation of atopic asthma, both childhood-onset and adult-onset. Development of CRS and asthma seem to involve several triggering factors, such as microbiome dysbiosis, which together with host barrier immunity leads to development and aggravation of the disease. However, it is not fully understood whether microbiome dysbiosis is a primary or secondary event. Microbiota changes during development, aging, sporadic events, treatment, between anatomic compartments, and between individuals. Well-controlled studies, and the latest methodology are mandatory to identify putative causal relationships between functionally active microbiota and chronic airway diseases. Bronchial epithelial sampling is more complicated, whereas nasal epithelial sampling can be performed easily, and with little harm. Genome-scale experiments and genome-environmental interaction analyses are important approaches when searching for pathways of chronic upper airway diseases.

## Author Contributions

All authors participated in writing of this article and critically reviewed the final article text.

## Conflict of Interest

ST-S has acted as paid consultant for ERT and Roche Products. RK has acted as a consultant for Sanofi-Regeneron, GSK, Genentech, Astellas, Lyra Therapeutics. All these are outside the submitted work. The remaining authors declare that the research was conducted in the absence of any commercial or financial relationships that could be construed as a potential conflict of interest.
